# Complete response to Mirvetuximab Soravtansine in platinum-resistant recurrent ovarian cancer: a case report

**DOI:** 10.1186/s13048-025-01628-z

**Published:** 2025-03-06

**Authors:** Yong Wu, Lingfang Xia, Chunyan Song, Xiaojun Chen, Xiaohua Wu

**Affiliations:** Department of Gynecologic Oncology, Fudan University Shanghai Cancer Center, Shanghai, China

## Abstract

Ovarian cancer, colloquially termed the "king of gynecological cancers," presents significant diagnostic and therapeutic challenges due to its covert nature. It ranks as the deadliest gynecologic malignancy with a disheartening 5-year survival rate below 40%. Standard therapeutic protocols for newly diagnosed patients encompass cytoreductive surgery followed by neoadjuvant or adjuvant platinum-based chemotherapy. Despite initial chemotherapeutic responses, recurrence is common, affecting up to 80% of patients, with nearly all developing eventual resistance to chemotherapy regimens. This case report highlights an Aisan patient with ovarian cancer, who exhibited tolerance, recurrence, and progression after several prior lines of treatment. The application of Mirvetuximab Soravtansine, facilitated by positive FRα expression identified through IHC analysis, notably reduced tumor lesions and CA125 levels, achieving a complete response and maintaining low CA125 levels during treatment, underscoring its efficacy in treating platinum-resistant recurrent ovarian cancer.

## Introduction

Ovarian cancer ranks as the deadliest gynecologic malignancy with a disheartening 5-year survival rate below 40%, posing substantial treatment challenges, particularly in cases of platinum-resistant recurrent ovarian cancer (PROC), where therapeutic options are severely limited [1, 2]. Current standard of care for these patients typically consists of primary cytoreductive surgery and platinum-based chemotherapy, followed by observation or maintenance treatment with bevacizumab, a poly (adenosine diphosphate [ADP]-ribose) polymerase inhibitor (PARPi), or combination of bevacizumab plus PARPi [3–6]. The objective response rates (ORR) for these therapies range merely from 4%−13%, with short-lived benefits, median progression-free survival (PFS) spanning only 2–5 months, and median overall survival (OS) less than one year [3–6]. While Bevacizumab combinations may enhance ORR, PFS, and OS, outcomes remain suboptimal, with ORR under 30% and median OS less than eighteen months. Despite the advent of novel therapies such as PARP inhibitors and antiangiogenic agents, which delay progression in platinum-sensitive recurrent cases, their efficacy remains limited in platinum-resistant populations. Similarly, extensive clinical trials of immunotherapies like checkpoint inhibitors (e.g., anti-PD-1/PD-L1 monoclonal antibodies) have not demonstrated clinical benefits in ovarian cancer, highlighting a profound unmet medical need in this patient cohort [3].

Recent decade has seen a new treatment strategy named as antibody–drug conjugates (ADCs). ADCs typically comprise a monoclonal antibody targeting antigens specific to tumor cell surfaces, linked to a potent cytotoxic agent. The folate receptor α (FRα), also known as FOLR1 or folate-binding protein, is a cysteine-rich glycoprotein pivotal in tumor growth and metastasis, mediating folate transport via receptor-endocytosis. Upon folate binding, FRα initiates crucial intracellular signaling, regulating pathways such as JAK-STAT3 and ERK1/2, and promoting oncogene expression within the nucleus [7, 8]. Predominantly expressed in epithelial-derived tumors like ovarian, breast, and lung cancers, FRα's overexpression in 76%−89% of epithelial ovarian cancers, especially absent in normal tissues, positions it as an optimal therapeutic target [9]. Immunohistochemistry (IHC) is the gold standard for the qualitative assessment of FOLR1 (FRα) in tumor tissues/lesions. Generally, based on the results of FOLR1 IHC staining, a PS2 + scoring system is employed (where tumor cells exhibit moderate intensity 2 + and/or strong 3 + membrane staining proportions) to determine the expression level of FRα, with a staining proportion of ≥ 75% indicating high expression, 50%−74% indicating moderate expression, and < 50% indicating low expression.

Mirvetuximab Soravtansine (MIRV), the only approved ADC targeting FRα, comprises a humanized anti-FRα monoclonal antibody (M9346A), a cleavable linker (sulfo-SPDB), and a microtubule inhibitor (DM4). It precisely targets FRα-positive tumor cells, leading to cell cycle arrest and apoptosis via DM4-mediated microtubule disruption [10–12]. Results from the phase III, single-arm SORAYA study confirmed an ORR of 32.4% (95% CI, 23.6–42.2), a median DOR of 6.9 months (95% CI, 5.6–9.7), and a DCR of 51% in FRɑ-positive patients with PROC. Median PFS was 5.5 months (95% CI, 3.8–6.9) as assessed by blinded independent central review and median OS was 15.0 months (95% CI, 11.5–18.7), demonstrating a clear clinical benefit. In November 2022, based on data from the SORAYA study [12], MIRV received accelerated approval from the FDA for the treatment of adult patients with FRα positive, platinum-resistant epithelial ovarian, fallopian tube, or primary peritoneal cancer, who have received one to three prior systemic treatment regimens.

Results from the Phase III global multicenter, confirmatory, open-label, randomized, controlled MIRASOL study showed that MIRV showed a significant benefit over chemotherapy with respect to progression-free and overall survival in patients with FRα-positive PROC, reducing the risk of disease progression/death by 35% (mPFS: 5.62 versus 3.98 months, HR = 0.65, P < 0.0001) and the risk of all-cause mortality by 33% (mOS: 16.46 vs 12.75 months, HR = 0.67, P = 0.0046). At the same time, compared with chemotherapy, MIRV can significantly improve ORR (42.3% versus 15.9%) and CA125 response rate (58% versus 30.3%), and the DCR (80.2% vs 56.2%) [13]. Fifty-six percent of patients had ocular adverse events after receiving MIRV treatment, of which 14% had grade 3 or higher ocular adverse events, almost all ocular AEs at follow-up were resolved to grade 0 or 1, and only four patients discontinued MIRV owing to ocular adverse events. No permanent ocular sequelae had been reported.

Here, we describe a case of 76-year-old Asian woman with platinum-resistant recurrent ovarian cancer, characterized by high FRα expression (95%, 3 + intensity), who achieved a complete response following treatment with Mirvetuximab Soravtansine after undergoing multiple prior therapies.

### Case presentation

**Fig. 1:**
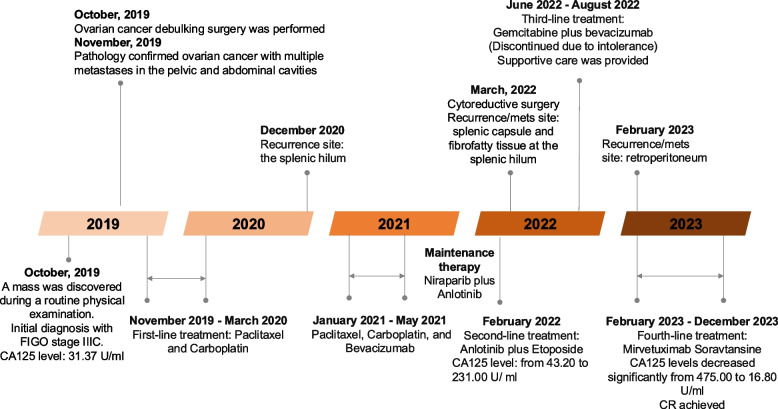
Overview of the treatment protocol

On October 8, 2019, a 76-year-old female presented with a right pelvic mass discovered during routine screening, showing a CA125 level of 31.37 U/ml, human epididymis protein 4 at 4170.5 pmol/L, and a postmenopausal ROMA index of 45.4%. The initial diagnosis was right ovarian cancer with suspected metastases to the interstitial region between the liver and kidney, classified as FIGO stage IIIC (Fig. 1). She underwent extensive debulking surgery on October 15, 2019, at Fudan University Shanghai Cancer Center, achieving an R0 resection with clean margins. The pathological findings in November 2019, confirmed poorly differentiated adenocarcinoma of the right ovary, measuring 4*1.8*1.5 cm, with involvement of the right fallopian tube and extensive metastasis in the pelvic and abdominal cavities. The patient received the first-line treatment with paclitaxel and carboplatin, completing six cycles from November 11, 2019, to March 11, 2020.

Follow-up imaging with an upper abdominal MRI conducted on December 2, 2020, revealed nodular formations at the splenic hilum, measuring 9 × 9 mm, suggestive of a potential metastatic origin. Additionally, multiple smaller nodules within the spleen were identified, raising suspicion of similar metastatic involvement. Treatment was resumed from January to May 2021 with a combination of paclitaxel, carboplatin, and bevacizumab for six cycles, followed by maintenance therapy using niraparib and anlotinib. A significant reduction in the splenic hilum lesion to 2.5mm was observed in an MRI on June 11, 2021, with other minor splenic lesions also diminishing or resolving.

An escalation in CA125 to 43.20 U/ml was noted on January 21, 2022, and a subsequent PET/CT on February 22, 2022, suggested reactivation of the tumor at the splenic hilum. After two weeks of the second-line treatment with anlotinib and etoposide (2 capsules daily for 2 weeks, followed by a 1-week break), CA125 levels increased to 231.00 U/ml. Imaging on March 17, 2022, revealed a low-density shadow under the splenic capsule with heightened FAPI uptake, slightly expanded from the previous FDG PET scan, suggesting the presence of active residual tumor. This led to a second debulking surgery on March 28, 2022, including splenectomy, partial diaphragm resection, and resolution of complex intestinal adhesions, achieving R0 resection. Specimens submitted for pathology examination included three firm, grayish-white nodules, measuring 0.2–1.4 cm in diameter, located near surgical suture sites and along the splenic capsule. An additional 0.6 cm grayish-white nodule was identified in the splenic hilum region, where the right hemidiaphragm was also involved, showing abundant psammoma body formation. A separate 2.0 * 1.0 * 0.4 cm tumor mass was also submitted. Another nodule (3.0 * 1.8 * 1.1 cm) from the splenocolic ligament was found to be a fat necrosis nodule with liquefaction. Pathology confirmed recurrent/metastatic adenocarcinoma within the splenic capsule and fibrofatty tissue at the splenic hilum. Immunohistochemical staining revealed partial positivity for ER, p16, and IMP3, while negativity for PR, HNF-1, and Napsin A. Additionally, the tumor showed positive staining for p53, WT1, and PAX-8, with a proliferation index (Ki-67) of approximately 30%. HER2 expression was weakly positive.

The patient underwent the third-line treatment with gemcitabine (1200 mg on days 1, 8, and 15) and bevacizumab (400 mg on days 1 and 15) from June to August 2022; however, the treatment was discontinued due to intolerance. Supportive care was provided, including liver protection, immune enhancement with thymalfasin and traditional Chinese medicine. By February 2023, a significant rise in CA125 to 555.10 U/ml was recorded, and PET/CT imaging revealed multiple new soft tissue density masses in the retroperitoneum, consistent with tumor infiltration.

**Fig. 2:**
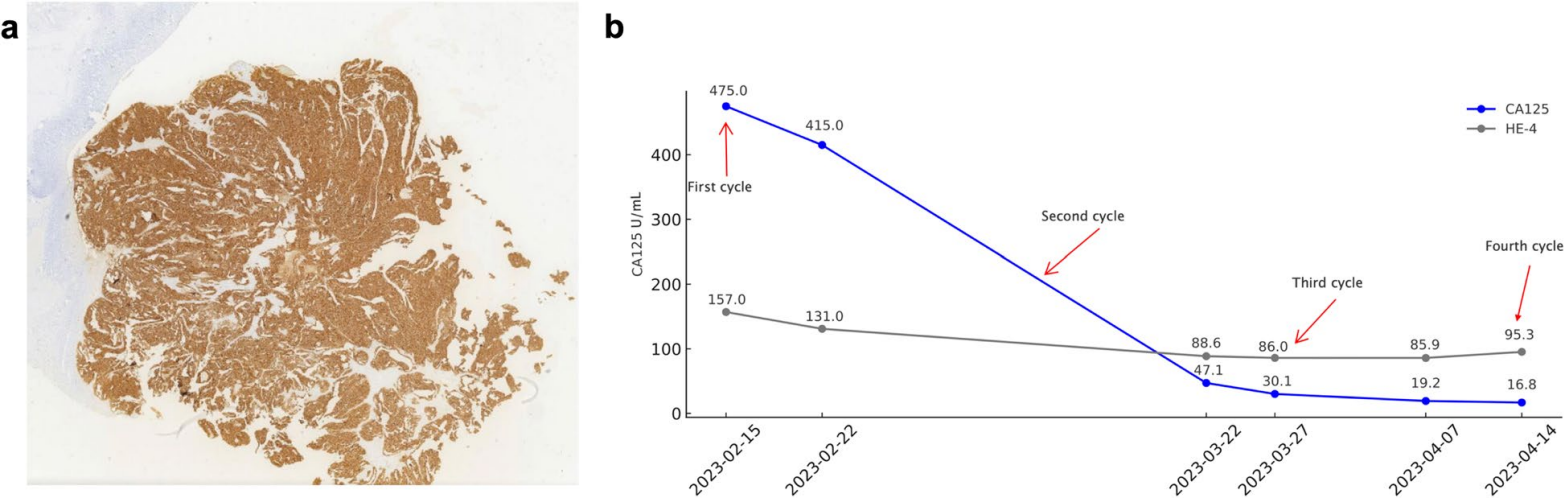
**a**) FRα staining **b**) Changes in CA125 levels during treatment with mirvetuximab soravtansine monoclonal antibody

On February 13, 2023, the patient underwent FRα expression testing using a folate test kit provided by a third-party testing center through immunohistochemical methods, with results indicating: FRα positive (tumor cell membrane positivity ratio 95%, staining intensity 3 +) **(**Fig. 2a**).** Consequently, the patient commenced the fourth-line of treatment with mirvetuximab Soravtansine monoclonal antibody on February 15, 2023, receiving a total of four treatments by April 19, 2023. MIRV, as a special medication, requires adherence to specific handling procedures. Firstly, due to the potential ocular adverse reactions associated with MIRV treatment, preventive ocular care is necessary, including prior ophthalmic examinations and premedication (artificial tears and corticosteroids). Additionally, premedication should be administered before each MIRV dose to reduce the incidence and severity of infusion-related reactions (IRR), nausea, and vomiting. The recommended dosing for MIRV is 6 mg/kg, calculated based on the adjusted ideal body weight (AIBW) of 54.8 kg for this patient, resulting in a dose of 329 mg administered every three weeks (Q3W), with a treatment cycle of 21 days, via intravenous infusion, until disease progression or the occurrence of intolerable toxicity. During the treatment period, a significant decrease in CA125 levels was observed, dropping from 475.00 U/ml before treatment to 16.80 U/ml within two months, and subsequently maintaining a steady decline, returning to normal ranges after two treatments as illustrated in Fig. 2b.

**Fig. 3:**
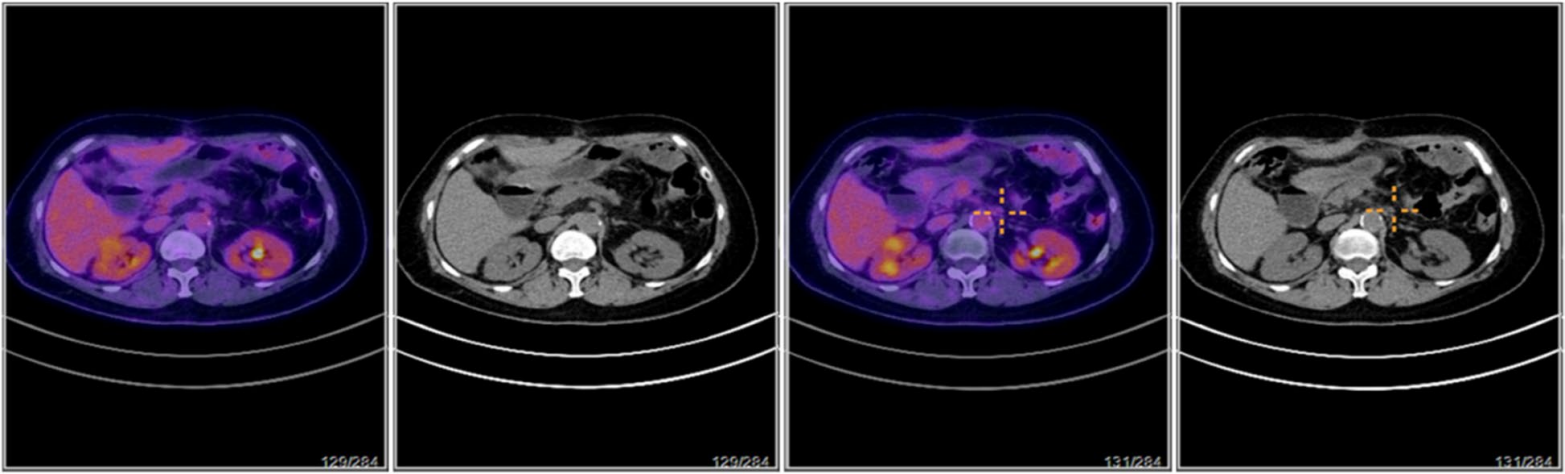
PET/CT Follow-up Examination on April 17, 2023

A follow-up PET/CT scan on April 17, 2023 (Fig. 3) indicated that post-surgery for ovarian cancer metastases in the spleen, a small retroperitoneal lymph node appeared with mildly increased FDG metabolism, showing a maximum SUV of approximately 1.8. Compared to the PET scan on January 31, 2023, the lesion had significantly reduced in size, with a decreased maximum SUV, suggesting changes due to treatment. Additionally, a high-density shadow under the left diaphragm next to the splenic surgical area was noted, with mildly increased FDG metabolism but a reduced maximum SUV value of approximately 2.0 from prior evaluations. No significant increases in FDG metabolism were observed elsewhere in the body, including the brain. The patient achieved a complete response according to oncological assessment. During the entire treatment period, the patient's state was good, and most adverse events were of low severity, mainly including blurred vision of grade 1. Subsequent treatments with mirvetuximab Soravtansine continued until December 2023 when they were halted due to pneumonia, during which CA125 levels fluctuated between 10.1 and 12.3, maintaining a low level, with the tumor in continued remission.

## Discussion

Effective treatments for patients with PROC remain elusive, highlighting an urgent need for new therapeutic strategies. Previous clinical trials have demonstrated the efficacy and safety of MIRV, yet certain limitations persist. Notably, most participants in these pivotal trials were from non-Asian populations, leaving a gap in efficacy data for Chinese patients. In the SORAYA trial [12], only two Asian patients were included, and in the MIRASOL trial [13], there were only 28; neither study provided detailed response outcomes for these patients. Here, we present the case of an elderly Asian female with heavily pretreated PROC who achieved a complete response to MIRV after multiple prior regimens. To our knowledge, this is the first documented report of a heavily pretreated Asian patient attaining a complete response to MIRV. This case report offers a more representative picture of MIRV’s effectiveness and safety in real-world practice, providing valuable insights for clinical decision-making.

FRα prominently overexpressed in many malignant tumors, especially ovarian cancer, has emerged as a promising target for the development of anticancer therapies. Current therapeutic strategies targeting FRα encompass protein antibodies and ADCs, with a variety of approaches such as monoclonal antibodies, ADCs, FRα-specific CAR-T cells, vaccines, and small molecules being explored. Advances in ADC technology have significantly propelled the development of FRα-targeting ADCs. Mirvetuximab soravtansine is specifically sanctioned for treating platinum-resistant ovarian cancer. Other FRα ADCs, like CBP-1008, STRO-002, MORAb-202, and AZD5335, have also demonstrated substantial antitumor efficacy in preclinical settings [14–16], supporting its clinical application.

ADCs are a class of biopharmaceutical drugs, typically being composed of a monoclonal antibody covalently attached to a cytotoxic drug, thus designed as targeted therapies [17]. Early clinical trials suggest that ADC monotherapy may be particularly effective in targeting tumor groups that exhibit resistance. Emerging evidence reveals that tumors may circumvent ADC activity through mechanisms such as downregulation of target antigens, altered internalization and processing of ADCs (which involves changes in intracellular transport pathways or lysosomal degradation of the drug), and development of resistance to the cytotoxic payload. Although ADC combination therapies have been investigated, the effectiveness of first-generation ADC combinations has been limited, possibly due to factors like non-specific target expression leading to adverse effects in healthy tissue, overlapping toxicities, ineffectiveness in various tumors, and undefined immune mechanisms.

In recent advancements, the structural design and clinical applications of ADCs have achieved significant improvements, yielding better outcomes across various tumor types. The fundamental design of ADCs, which involves the covalent attachment of cytotoxic drugs to tumor-specific antibodies, has notably reduced the adverse effects associated with conventional chemotherapy. Nevertheless, the clinical use of ADCs continues to confront issues with off-target toxicity, target-related toxicity, and unpredictable adverse reactions. In ovarian cancer, the majority of observed toxicities are off-target, including peripheral neuropathy, gastrointestinal toxicity, hematological toxicity, and ocular toxicity. Notably, over 90% of adverse events in clinical settings involving ADCs are linked to the cytotoxic payload. Different ADCs carrying the same cytotoxic agents tend to exhibit similar profiles of adverse reactions; those containing microtubule inhibitors frequently induce ocular and hepatic toxicities and thrombocytopenia, while ADCs with topoisomerase I inhibitors like DXd and SN-38 are often associated with hair loss, diarrhea, and neutropenia—a consequence typically attributed to the effects of microtubule toxins.

Although the mechanisms behind ADC-induced ocular toxicity remain unclear, the absence of FRα receptors in ocular tissues suggests off-target effects on the corneal epithelium are likely responsible. Prior studies have shown that payloads like DM4 can cause reversible ocular toxicities, including blurred vision, dry eyes, and corneal lesions. Efforts are underway to develop strategies for the prevention and treatment of ocular toxicity and neuropathy. Overall, the toxicities associated with ADCs are manageable, with minimal hematological impact and reduced gastrointestinal side effects. Given the dose-dependent nature of ADC adverse reactions, dose optimization strategies based on patient weight and pharmacokinetics are crucial to avoid overdosing and mitigate unnecessary adverse effects, and dose adjustments during treatment are guided by patient response to maximize therapeutic efficacy and minimize side effects [18, 19].

The integration of ADCs with immunotherapy, targeted therapy, and chemotherapy signals a significant direction for future oncological strategies. The use of ADCs in conjunction with immune checkpoint inhibitors can sustain an activated immune state within the tumor microenvironment via various immunomodulatory mechanisms, including the reprogramming of macrophages, the generation of T memory cells, the induction of enhanced PD-L1 expression, and the activation of dendritic and T cells. The cell death mediated by ADCs may provoke immunogenic cell death (ICD), thereby fostering anti-tumor immune responses and allowing immune effector cells to identify "cold" tumors. This induction of ICD underscores the efficacy of combining ADCs with immune checkpoint inhibitors [20, 21].

Furthermore, the concomitant use of ADCs with tyrosine kinase inhibitors (TKIs) permits the blocking of multiple targets concurrently with greater specificity, thereby inhibiting downstream signaling pathways more effectively. Certain TKIs have also demonstrated the ability to modulate surface antigens, increasing tumor cell susceptibility to ADCs and amplifying their activity. The addition of anti-angiogenic agents, such as bevacizumab, to ADC therapy may enhance ADC delivery to tumor tissues by normalizing tumor vasculature, thus intensifying their cytotoxic effects [20, 22]. Specifically, according to FORWARDII study, mirvetuximab Soravtansine combined with bevacizumab achieved an ORR of 44% and 48% in the PROC and PSOC populations, respectively, and a median PFS of 8.2 and 9.6 months, respectively, and regardless of the high, medium or low expression of FRα, combined treatments can show great benefit, showing stronger anti-tumor activity than a single drug, while maintaining a controllable safety profile [20, 22].

Combining ADCs with chemotherapy can synergistically amplify therapeutic efficacy through mechanisms such as inducing cell cycle arrest and inhibiting DNA repair. For instance, the combination of mirvetuximab soravtansine and carboplatin has yielded an ORR of 71% and a median PFS of 9.6 months in the PSOC population, indicating superior outcomes when DNA damaging agents and microtubule inhibitor ADCs are used together. However, it is crucial to monitor for cumulative toxicities such as gastrointestinal reactions or hematologic toxicity [22]. Additionally, chemotherapy drugs can influence the expression of surface antigens targeted by ADCs, either enhancing antigen–antibody binding or promoting their degradation, thereby altering the dynamic balance of target antigens and further enhancing the activity of ADCs [20].

A limitation of this case report is the unavailability of homologous recombination deficiency (HRD) testing and BRCA mutation analysis (both germline and somatic). Additionally, the follow-up period in this report is relatively short, which limits the ability to fully assess the long-term efficacy and durability of the response to mirvetuximab soravtansine.

In conclusion, this case highlights Mirvetuximab Soravtansine as a promising therapeutic option for PROC and underscores its potential applicability in underrepresented populations, providing valuable insights into real-world practice.

## Data Availability

No datasets were generated or analysed during the current study.
